# Bone microarchitectural analysis using ultra-high-resolution CT in tiger vertebra and human tibia

**DOI:** 10.1186/s41747-019-0135-0

**Published:** 2020-01-28

**Authors:** Ryota Inai, Ryuichi Nakahara, Yusuke Morimitsu, Noriaki Akagi, Youhei Marukawa, Toshi Matsushita, Takashi Tanaka, Akihiro Tada, Takao Hiraki, Yoshihisa Nasu, Keiichiro Nishida, Toshifumi Ozaki, Susumu Kanazawa

**Affiliations:** 1 Department of Radiology, Okayama University Medical School, 2-5-1 Shikatacho, Kitaku, Okayama, 700-8558 Japan; 2 Intelligent Orthopaedic System Development, Okayama University Medical School, 2-5-1 Shikatacho, Kitaku, Okayama, 700-8558 Japan; 3 Devision of Radiology, Medical Support Department, Okayama University Hospital, 2-5-1 Shikatacho, Kitaku, Okayama, 700-8558 Japan; 4 Medical materials for musculoskeletal reconstruction, Okayama University Medical School, 2-5-1 Shikatacho, Kitaku, Okayama, 700-8558 Japan; 5 Orthopaedic Surgery, Okayama University Medical School, 2-5-1 Shikatacho, Kitaku, Okayama, 700-8558 Japan

**Keywords:** Osteoporosis, Bone density, Tomography (x-ray computed), X-ray microtomography

## Abstract

**Background:**

To reveal trends in bone microarchitectural parameters with increasing spatial resolution on ultra-high-resolution computed tomography (UHRCT) *in vivo* and to compare its performance with that of conventional-resolution CT (CRCT) and micro-CT *ex vivo*.

**Methods:**

We retrospectively assessed 5 tiger vertebrae *ex vivo* and 16 human tibiae *in vivo*. Seven-pattern and four-pattern resolution imaging were performed on tiger vertebra using CRCT, UHRCT, and micro-CT, and on human tibiae using UHRCT. We measured six microarchitectural parameters: volumetric bone mineral density (vBMD), trabecular bone volume fraction (bone volume/total volume, BV/TV), trabecular thickness (Tb.Th), trabecular number (Tb.N), trabecular separation (Tb.Sp), and connectivity density (ConnD). Comparisons between different imaging resolutions were performed using Tukey or Dunnett T3 test.

**Results:**

The vBMD, BV/TV, Tb.N, and ConnD parameters showed an increasing trend, while Tb.Sp showed a decreasing trend both *ex vivo* and *in vivo*. *Ex vivo*, UHRCT at the two highest resolutions (1024- and 2048-matrix imaging with 0.25-mm slice thickness) and CRCT showed significant differences (*p* ≤ 0.047) in vBMD (51.4 mg/cm^3^ and 63.5 mg/cm^3^ *versus* 20.8 mg/cm^3^), BV/TV (26.5% and 29.5% *versus* 13.8 %), Tb.N (1.3 l/mm and 1.48 l/mm *versus* 0.47 l/mm), and ConnD (0.52 l/mm^3^ and 0.74 l/mm^3^ *versus* 0.02 l/mm^3^, respectively). *In vivo*, the 512- and 1024-matrix imaging with 0.25-mm slice thickness showed significant differences in Tb.N (0.38 l/mm *versus* 0.67 l/mm, respectively) and ConnD (0.06 l/mm^3^ *versus* 0.22 l/mm^3^, respectively).

**Conclusions:**

We observed characteristic trends in microarchitectural parameters and demonstrated the potential utility of applying UHRCT for microarchitectural analysis.

## Key points

- The improvement of spatial resolution with ultra-high-resolution whole-body computer tomography (CT) has the potential to improve bone microarchitectural analysis.
- Bone microarchitectural analysis with a whole-body CT can be used to evaluate osteoporosis at any bone site and can reuse imaging data that were previously obtained for other purposes using only 10-mm-range volume data.
- The identified trends of microarchitectural parameters at different spatial resolutions can be used as precise indicators of the performance of CT and thereby inform their further development.

## Background

Osteoporosis is a skeletal disorder characterised by compromised bone strength that predisposes patients to an increased risk of fractures [1]. Considering that older adults are especially prone to developing this condition, and because older adults are constituting an increasingly greater proportion of the populations in developing countries, osteoporosis is becoming a pressing public health concern [2]. Various methods have therefore been established to identify patients at high-risk for osteoporotic fractures, as well as to initiate appropriate therapeutic measures before osteoporosis-associated fractures occur. Imaging methods used for this purpose are based on the measurement of bone mineral density (BMD) with dual-energy x-ray absorptiometry (DXA) [3].

DXA provides information regarding the areal BMD of the lumbar spine (L1–L4) and femoral neck. Areal BMD measurements obtained with DXA are currently considered to be the most significant predictors of fracture risk; however, BMD only indicates the bone mass and does not account for all aspects of fractures [4]. Additionally, DXA is a multistep procedure that requires demographic information, patient positioning, correct image analysis, and artefact identification. Errors have the potential to occur at any step and have been reported in more than 90% of DXA examinations [5]. Hence, clinicians have sought other methods to assess bone quality, yielding new concepts that encompass BMD and several other bone characteristics, such as apatite crystallisation, collagen properties, and trabecular microarchitecture [6].

Independent of BMD [7], bone microarchitecture is reported as a key determinant of bone strength, and its deterioration has been included in the World Health Organization definition of osteoporosis [2]. Several novel methods of assessing bone microarchitecture, including the trabecular bone score, bone strain index obtained with DXA [8, 9], and high-resolution peripheral quantitative computed tomography (HR-pQCT), have made it possible to assess volumetric bone mineral density (vBMD) and the microarchitecture of the radius and tibia [10]. High-resolution trabecular bone imaging yields additional information beyond the areal BMD measurements obtained from DXA to predict bone strength [11], whereas HR-pQCT is limited to peripheral skeletal sites. On the other hand, although three reports have described microarchitectural analyses of whole-body CT obtained with conventional-resolution CT (CRCT) [12–14], no additional studies have suggested that the resolution of CRCT is critical for microarchitecture analysis.

Two previous reports have described the relationship between microarchitectural parameters and spatial resolution using different CT scanners. Specifically, the studies compared HR-pQCT with micro-CT across 17 radii from human cadaver specimens. Baum et al. [14] demonstrated the effect of voxel size on structural measures obtained from the trabecular and cortical bones; Baum et al. analysed trabecular bone structure parameters measured by a clinical multidetector CT in relation to those from HR-pQCT for 14 spinal segments from human cadavers.

Recently introduced in clinical settings [15], ultra-high-resolution CT (UHRCT) is a type of whole-body CT that features 128 detector rows of 0.25 mm width in a 2048 × 2048 matrix, providing more than a 2-fold increase in spatial resolution. Currently, UHRCT has yet to be compared with other CT scanners; hence, the effects of changing the slice thicknesses and matrix in UHRCT on the patterns of microarchitectural parameters remain unknown.

This study aimed to reveal the trends in bone microarchitectural parameters based on the increase in spatial resolution by three different CT scanners for *ex vivo* assessments, matrix numbers, and slice thicknesses on UHRCT for *in vivo* assessments. The secondary aim was to demonstrate the potential application of UHRCT for bone microarchitectural analysis relative to the performances of CRCT and micro-CT by revealing the trends of bone microarchitectural parameters. We hypothesised that whole-body CT imaging using UHRCT may be as advantageous for the analysis of bone microarchitecture as HR-pQCT, which is limited to peripheral sites.

## Methods

### Subjects

**Fig. 1:**
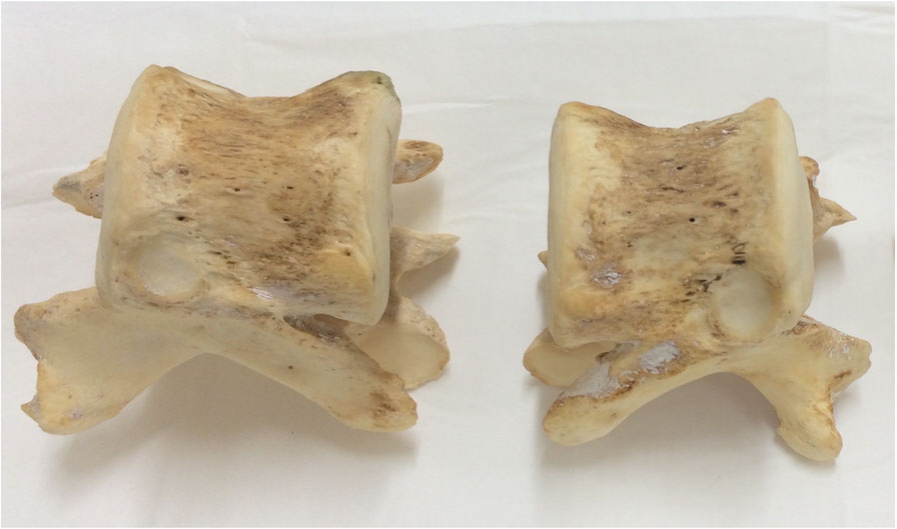
Examples of dry tiger vertebral bones used as *ex vivo* subjects

This study assessed 5 *ex vivo* tiger vertebrae and 16 human tibiae in vivo. We obtained dry tiger, rabbit, meerkat (*Suricata suricatta*), and tuna vertebrae in cooperation with the Zoological Park of Okayama City, Japan (Fig. 1). Among these, we chose tiger vertebrae as *ex vivo* subjects because they featured abundant trabeculae and possessed simple shape areas; these properties facilitate setting the regions of interest. Images of the same areas in tiger vertebrae were obtained using CRCT, UHRCT, and micro-CT *ex vivo*. The areas were categorised into 7 groups: tiger vertebra groups 1–7 (T1–T7). Group T1 underwent CRCT, groups T2–T6 underwent UHRCT with various matrix numbers and slice thicknesses, and group T7 underwent micro-CT (Table 1).

**Table 1:** Imaging conditions for the seven spatial resolution groups with different CT scanners, matrix numbers, and slice thicknesses for the tiger vertebra

Group	T1	T2	T3	T4	T5	T6	T7
CT scanner	CRCT	UHRCT	UHRCT	UHRCT	UHRCT	UHRCT	μCT
Matrix number	512	512	512	1024	1024	2048	2048
Slice thickness (mm)	0.5	0.5	0.25	0.5	0.25	0.25	0.06
Pixel value (μm)	350	150–350	150–350	150–350	150–350	150–350	60
Tube voltage (kVp)	120	120	120	120	120	120	50
Tube load (mA)	100	100	100	100	100	100	0.5
Field of view (mm)	120	120	120	120	120	120	120

**Fig. 2:**
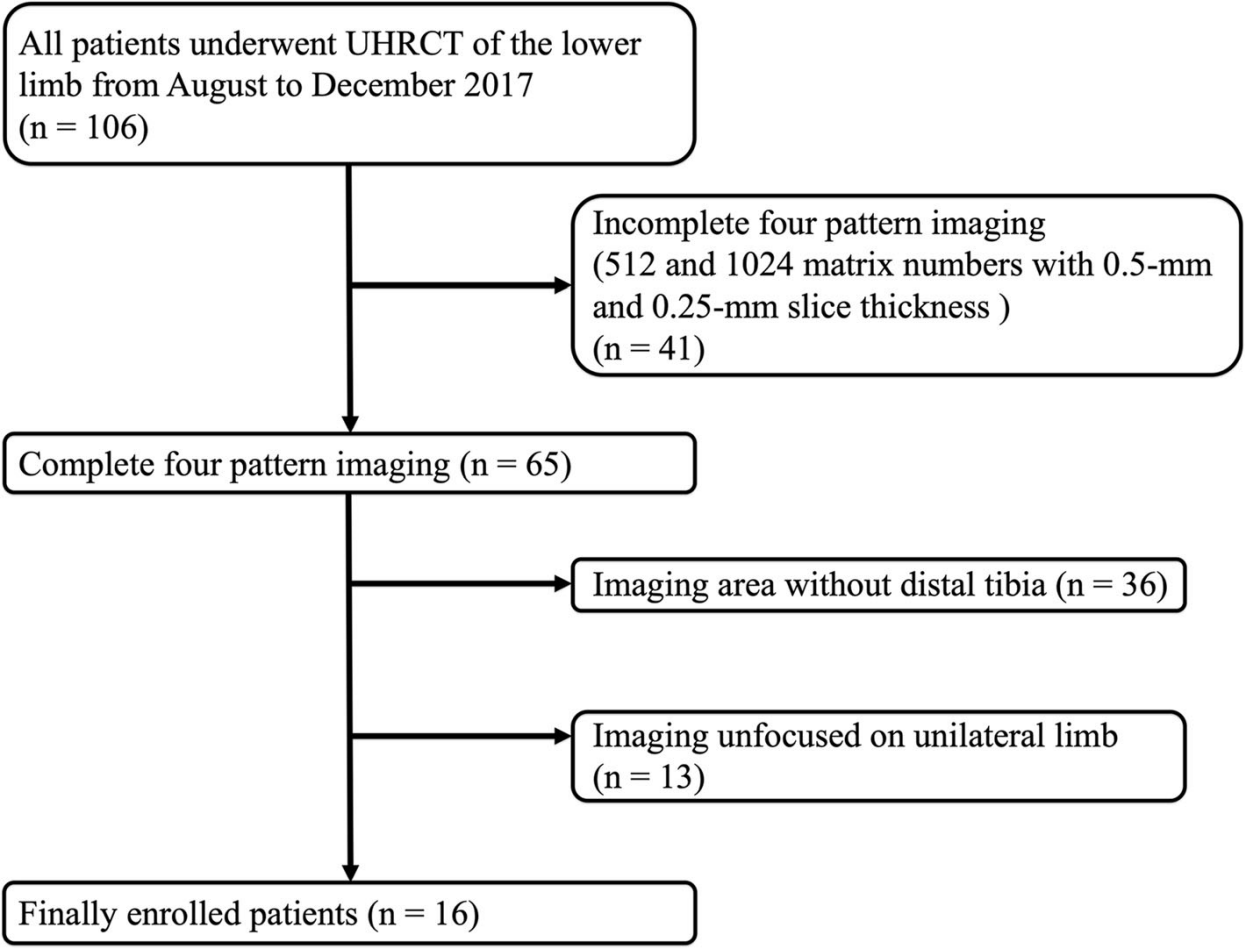
Patient selection flowchart. *UHRCT* Ultra-high-resolution computed tomography

We also assessed the human distal tibia *in vivo* because this region is a common site at which microarchitecture is analysed with HR-pQCT. We retrieved the data from 106 patients who underwent UHRCT for lower limb assessments from August to December 2017 at our institution (Fig. 2). We then identified 65 patients whose images were obtained with four combinations of matrix numbers (512 × 512 and 1024 × 1024) and slice thicknesses (0.5 mm and 0.25 mm). Among these 65 patients, we excluded 36 patients without distal tibia imaging data and 13 patients with images that did not focus on a unilateral limb. Finally, we included 16 patients (10 women). The findings were classified into 4 groups (human tibia groups 1–4: H1–H4) based on the matrix number and slice thickness used for UHRCT (Table 2). Ethical approval for the study protocol was obtained from the Institutional Research Ethics Board, and the requirement for informed consent was waived.

**Table 2:** Imaging conditions for the four spatial resolution groups with UHRCT with different matrix numbers and slice thicknesses for the human tibia

Group	H1	H2	H3	H4
CT scanner	UHRCT	UHRCT	UHRCT	UHRCT
Matrix number	512	512	1024	1024
Slice thickness (mm)	0.5	0.25	0.5	0.25
Pixel value (μm)	150–350	150–350	150–350	150–350
Tube voltage (kVp)	120	120	120	120
Tube load (mA)	101–160	101–160	101–160	101–160
Field of view (mm)	160–260	160–260	160–260	160–260

### Micro-CT

The small laboratory animal micro-CT system (LaTheta LCT-200; Aloka, Tokyo, Japan; Image field < 120 mm) is used for imaging objects set in small cases; therefore, we used the micro-CT scanner to access only the tiger vertebrae, not the distal human tibia. We substituted the micro-CT scan for HR-pQCT because there was no HR-pQCT system at our institution. The matrix number, slice thickness, and pixel value were 2048 × 2048, 0.06 mm, and 60 μm, respectively. The scan parameters were as follows: tube voltage 50 kVp, tube load 0.5 mAs, and field of view 120 × 120 mm (Table 1). A total of 150 slices were collected 10 mm from a reference line at the endplate of the caudal end.

### CRCT and UHRCT

Both CRCT and UHRCT are whole-body CT scanners that are generally used in clinical practice. CRCT (Aquilion One, Canon Medical Systems, Otawara, Japan) was only used to obtain *ex vivo* images of the tiger vertebrae (matrix 512 × 512; slice thickness 0.5 mm; pixel value 350 × 350 μm). Scan parameters were as follows: tube voltage 120 kVp, tube load 100 mAs, and field of view 120 × 120 mm (Table 1). The same imaging range as the micro-CT was used.

The UHRCT scanner (Aquilion Precision, Canon Medical Systems, Otawara, Japan) was used for both tiger vertebrae and human tibiae. For imaging the tiger vertebrae, we used three matrix values (512 × 512, 1024 × 1024, and 2048 × 2048), two slice thicknesses (0.5 mm, 0.25 mm), and pixel values from 150 × 150 μm to 350 × 350 μm. The scan parameters were as follows: tube voltage 120 kVp, tube load 100 mAs, and field of view 120 × 120 mm (Table 1). The imaging range corresponding to the micro-CT was collected. For imaging of the human tibiae, we used two matrix patterns (512 × 512, 1024 × 1024) and two slice thicknesses (0.5 mm, 0.25 mm). The scan parameters were as follows: tube voltage 120 kVp, tube load 101–160 mA, and field of view from 160 × 160 to 260 × 260 mm (Table 2). Consistent with previous studies, the image range was 9 mm and was obtained 22.5 mm from a reference line at the endplate of the distal tibia.

### Image analysis

Bone microarchitecture was calculated with a three-dimensional image analysis system (TRI/3D-BON; RATOC System Engineering, Tokyo, Japan) [16]. We measured vBMD by determining the linear attenuation values converted to hydroxyapatite mineral densities using a phantom for CRCT and UHRCT (B-MAS200; KYOTO KAGAKU, Kyoto, Japan) and for micro-CT (No6-U5D1mmH; RATOC System Engineering, Tokyo, Japan). Adaptive thresholds of vBMD to extract trabecular and cortical bone were 50 and 200 mg/cm^3^, respectively. The thresholds for human tibia were 210 mg/cm^3^ and 200–500 mg/cm^3^ for the trabecular and cortical bones, respectively. We measured the trabecular vBMD, trabecular bone volume fraction (bone volume/trabecular volume, BV/TV), trabecular thickness (Tb.Th), trabecular number (Tb.N), trabecular separation (Tb.Sp), and connectivity density (ConnD). BV/TV was calculated by dividing the trabecular bone volume by the entire marrow area volume, including the trabecular bone. Tb.Th and Tb.Sp were determined by filling maximal spheres into the structure according to a previously described method [17]. Tb.N was estimated as a trabecular bone number crossing the line perpendicular to the growing direction of vertebrae based on the plate model [18]. ConnD indicated the number of redundant connections between trabecular structures per unit volume.

### Statistical analysis

All statistical analyses were performed using SPSS software version 22 (SPSS, Inc., Chicago, IL, USA). Mean and standard deviation were calculated for all subjects. We compared the seven tiger vertebra groups (T1–T7) *ex vivo* for each bone microarchitectural parameter. The four *in vivo* human tibia groups (H1–H4) were compared in the same way. Levene’s test was used to analyse the distributions of numerical variables. All groups were compared using either the Tukey test or Dunnett T3 test, as appropriate. Two-tailed *p* values < 0.050 were considered to be statistically significant for all analyses.

## Results

### Subject characteristics

We studied 5 tiger vertebrae *ex vivo* and 16 human tibiae of patients who underwent UHRCT *in vivo*. The patients underwent UHRCT on account of having been diagnosed with diseases of the lower limb (osteoarthritis, 4 patients; rheumatoid arthritis and trauma, 2 patients each; and arthritis, bone metastasis, Langerhans cell histiocytosis, symptomatic accessory navicular bone, liposarcoma, intramuscular metastasis, plantar fasciitis, and cellulitis, 1 patient each). The mean age of the patients was 55.5 ± 22.4 years (range, 2–89 years). Each patient had a history of bisphosphonate and oral corticosteroid use, and 6 of the female patients were postmenopausal. No lesions were observed in the imaging areas for analysis of bone microarchitecture either *ex vivo* or *in vivo*. The CT dose index of ankle joint images for 12 patients was 7.1 ± 1.3 mGy. Further, it was 11.7 ± 0.6 mGy for the images from knee to ankle in 2 patients and 11.8 ± 1.1 mGy for the images from hip to the ankle in 2 patients.

### Comparisons between CRCT, UHRCT, and micro-CT in tiger vertebra *ex vivo*

**Fig. 3:**
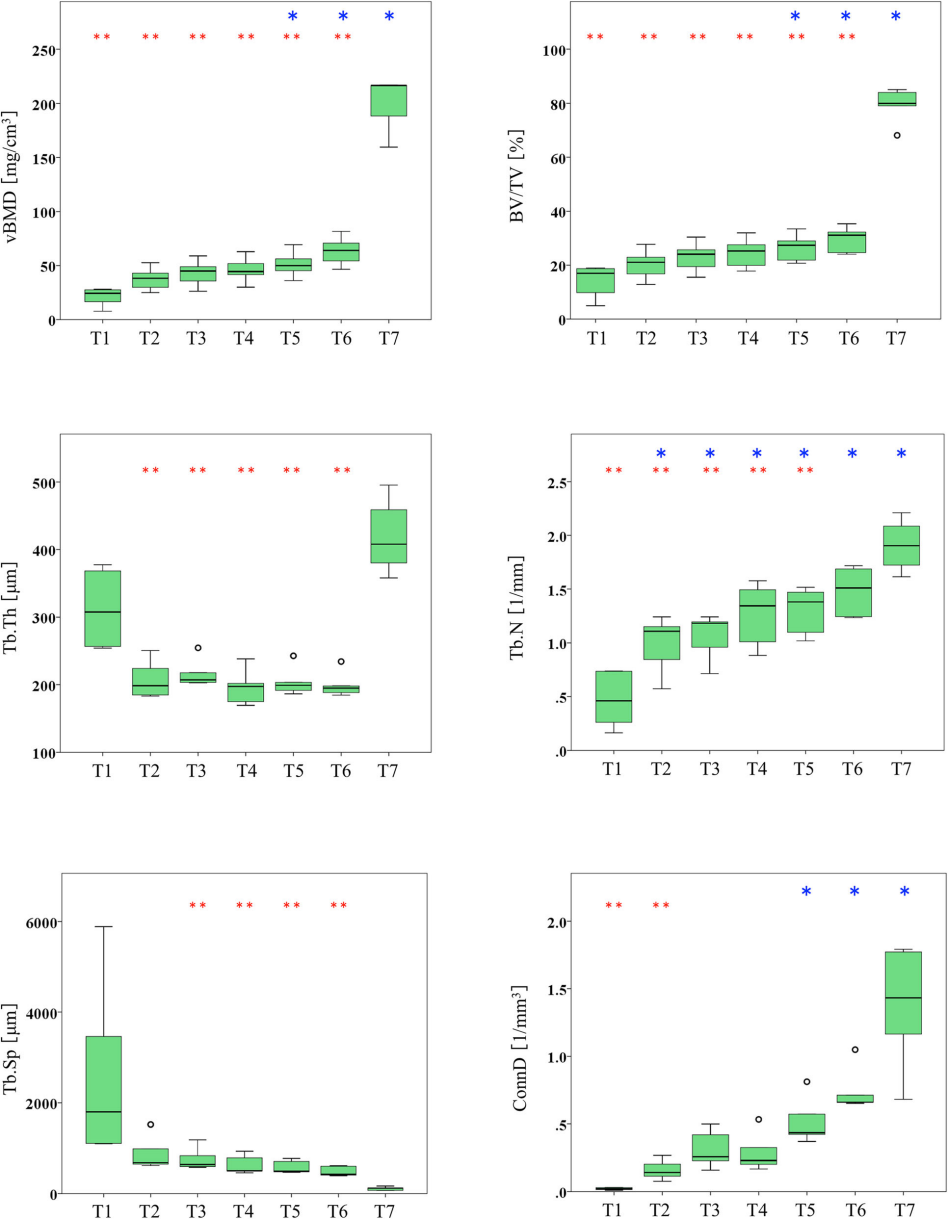
Box-and-whisker plots showing six bone microarchitectural parameters at seven different spatial resolution groups in tiger vertebra (T1 = conventional-resolution computed tomography (CT), matrix 512 × 512, slice thickness 0.5 mm; T2–T6 = ultra-high-resolution CT; T2, matrix 512 × 512, slice thickness 0.5 mm; T3, matrix 512 × 512, slice thickness 0.25 mm; T4, matrix 1024 × 1024, slice thickness 0.5 mm; T5, matrix 1024 × 1024, slice thickness 0.25 mm; T6 = matrix 2048 × 2048, slice thickness 0.25 mm; T7 = micro-CT, matrix 2048 × 2048, slice thickness 0.06) mm. *T1–T7* Tiger vertebra groups 1–7, *vBMD* × 2048 Volumetric bone mineral density, *BV/TV* Trabecular bone volume fraction, *Tb.Th* Trabecular thickness, *Tb.N* Trabecular number, *Tb.Sp* Trabecular separation, *ConnD* Connectivity density. Open circles indicate outliers. Asterisk (*) and double asterisk (**) indicate significant differences from the conventional-resolution CT group (T1) or micro-CT group (T7), respectively (*p* ≤ 0.047). Tukey or Dunnett T3 tests were used, as appropriate

The bone microarchitectural parameters are presented in Table 3 and Fig. 3. The vBMD and BV/TV values tended to increase with increases in resolution, and there were statistically significant differences between the micro-CT group (T7) and all of the UHRCT groups (T2–T6) (*p* ≤ 0.037 for both, Fig. 3). The two highest-resolution UHRCT groups (T5 and T6) showed significantly higher values than those obtained in CRCT (group T1) for vBMD (mean, 51.4 mg/cm^3^ and 63.5 mg/cm^3^ *versus* 20.8 mg/cm^3^, respectively) and BV/TV (26.5% and 29.5% *versus* 13.8%, respectively), as shown in Table 3. For Tb.Th, the values for all UHRCT groups (group T2–T6) were lower than those for CRCT (group T1) and micro-CT (group T7), with significant differences noted in comparison with the micro-CT values. Tb.N values showed an increasing trend with higher resolutions. There were statistically significant differences between the four UHRCT groups (group T2–T5) and micro-CT (group T7), as well as between all of the UHRCT groups (group T2–T6) and the CRCT (group T1), *i.e*., the value for the highest-resolution UHRCT group (group T6) was not significantly different from that for micro-CT (1.48 l/mm *versus* 1.91 l/mm). The Tb.Sp value tended to decrease with increasing resolution. The values for the four UHRCT groups (groups T3–T6) were higher than those for micro-CT (768 μm, 637 μm, 586 μm, and 489 μm *versus* 111 μm, respectively). ConnD values tended to increase with increases in resolution. The value for the lowest resolution UHRCT group (group T2) was significantly lower than that for micro-CT (0.16 l/mm^3^ *versus* 1.36 l/mm^3^). The values for the two highest-resolution UHRCT groups (groups T5 and T6) were significantly higher than that for CRCT (0.52 l/mm^3^ and 0.74 l/mm^3^ *versus* 0.02 l/mm^3^, respectively).

**Table 3:** Comparison of tiger vertebral microarchitectural parameters obtained with different CT scanners, matrix numbers, and slice thicknesses *ex vivo*

	Group						
	T1	T2	T3	T4	T5	T6	T7
CT scanner	CRCT	UHRCT	UHRCT	UHRCT	UHRCT	UHRCT	Micro-CT
Matrix number	512	512	512	1024	1024	2048	2048
Slice thickness (mm)	0.5	0.5	0.25	0.5	0.25	0.25	0.06
vBMD (mg/cm^3^)	20.8 ± 8.6	37.7 ± 10.9	42.9 ± 12.5	46.2 ± 12.2	51.4 ± 12.4	63.5 ± 13.7	199.6 ± 25.5
BV/TV (%)	13.8 ± 6.2	20.2 ± 5.7	23.1 ± 5.7	24.5 ± 5.7	26.5 ± 5.2	29.5 ± 4.9	79.2 ± 6.7
Tb.Th (μm)	312.8 ± 58.8	208.1 ± 28.8	217.1 ± 21.7	196.3 ± 27.2	204.6 ± 22.3	200 ± 19.9	420 ± 56.6
Tb.N (l/mm)	0.47 ± 0.26	0.98 ± 0.27	1.06 ± 0.22	1.26 ± 0.3	1.3 ± 0.22	1.48 ± 0.23	1.91 ± 0.24
Tb.Sp (μm)	2671 ± 2040	892 ± 380	768 ± 253	637 ± 210	586 ± 146	489 ± 109	111 ± 40
ConnD (l/mm^3^)	0.02 ± 0.01	0.16 ± 0.07	0.31 ± 0.14	0.29 ± 0.14	0.52 ± 0.17	0.74 ± 0.17	1.36 ± 0.46

Data are means ± standard deviations

*T1–T7* Tiger vertebra groups 1–7, *CRCT* Conventional resolution computed tomography, *UHRCT* Ultra-high-resolution computed tomography, *vBMD* Volumetric bone mineral density, *BV/TV* Trabecular bone volume fraction, *Tb.Th* Trabecular thickness, *Tb.N* Trabecular number, *Tb.Sp* Trabecular separation, *ConnD* Connectivity density

**Fig. 4:**
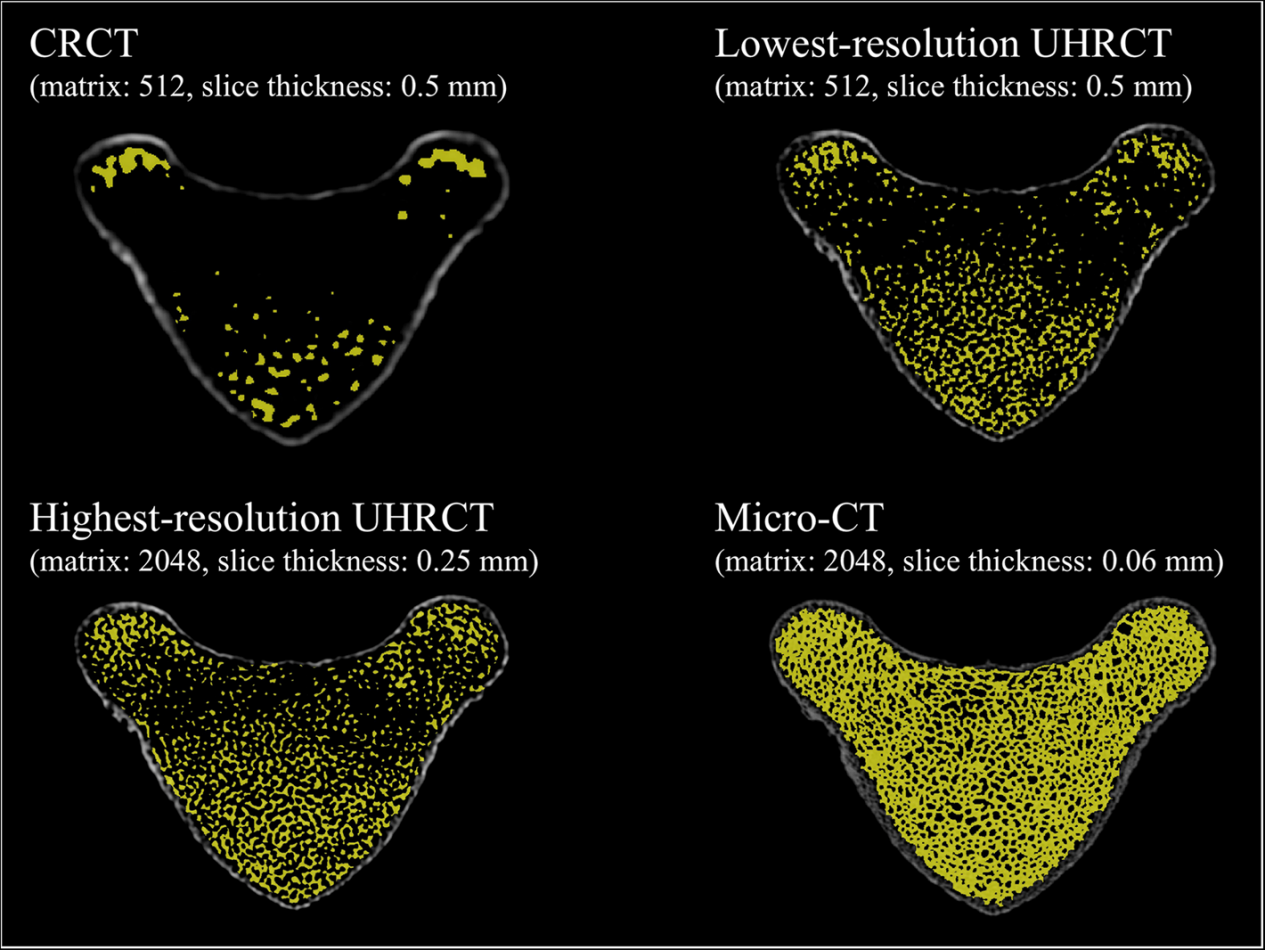
Representative extracted images of the trabecular bone in tiger vertebrae on CRCT, lowest-resolution (matrix 512 × 512, slice thickness 0.5 mm) and highest-resolution (matrix 2048 × 2048, slice thickness 0.25 mm) UHRCT, and micro-CT. *CRCT* Conventional-resolution computed tomography, *UHRCT* Ultra-high-resolution computed tomography

Extracted images that are representative of the trabecular bone for each CT scanner are shown in Fig. 4. These extracted images reveal that CRCT could not accurately detect the trabecular bone. The micro-CT image shows a more precise depiction than does the UHRCT image. Nevertheless, UHRCT preserved high visual quality of trabecular bone over the entire bone marrow area, which was visually different from the CRCT findings.

### Comparison of different matrix numbers and slice thicknesses on UHRCT for *in vivo* assessments of the human tibia

**Fig. 5:**
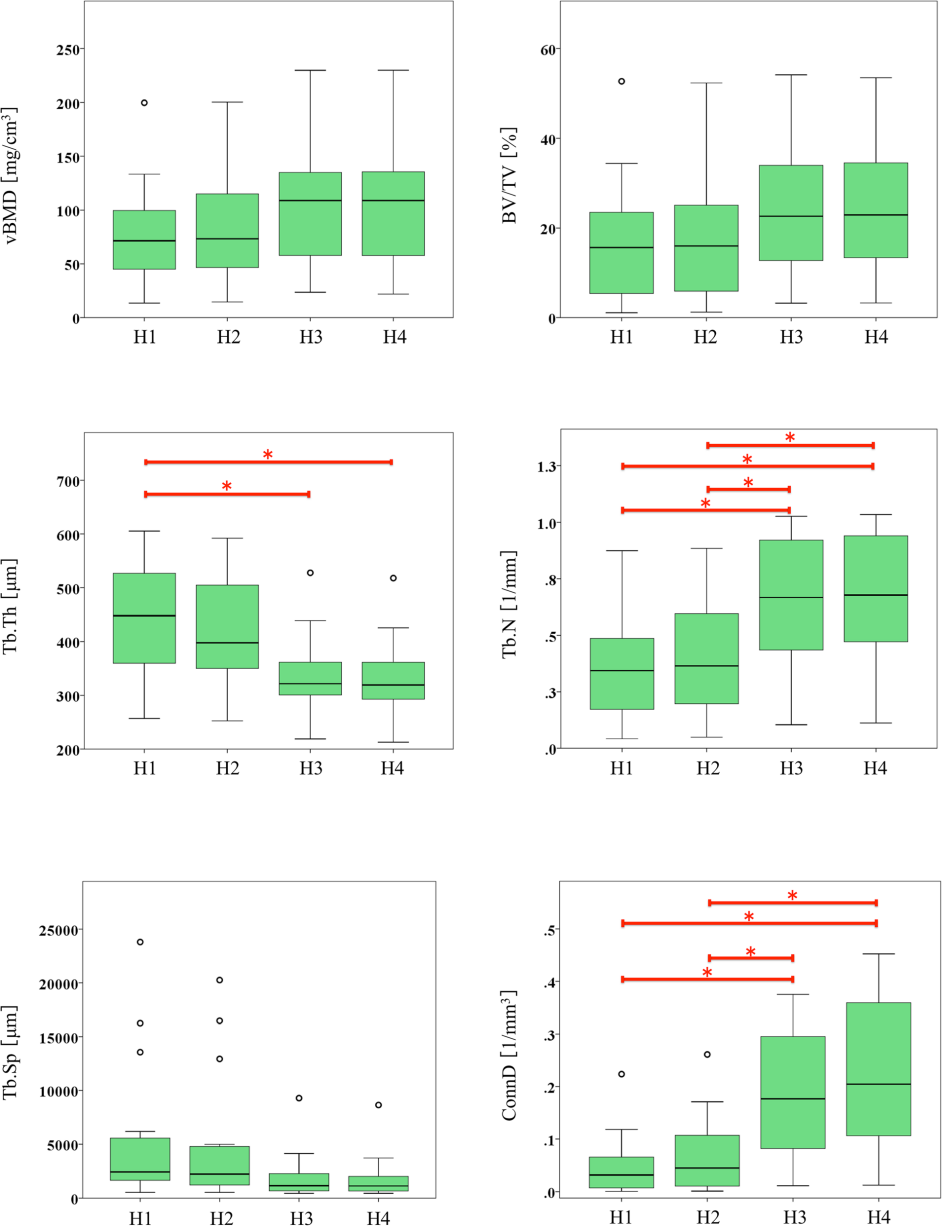
Box-and-whisker plots showing six bone microarchitectural parameters at four different spatial resolution groups on ultra-high-resolution CT in human tibia (H1, matrix 512 × 512, slice thickness 0.5 mm; H2, matrix 512 × 512, slice thickness 0.25 mm; H3, matrix 1024 × 1024, slice thickness 0.5 mm; H4, matrix 1024 × 1024, slice thickness 0.25 mm). *H1–H4* Human tibia group 1–4, *vBMD* Volumetric bone mineral density, *BV/TV* Trabecular bone volume fraction, *Tb.Th* Trabecular thickness, *Tb.N* Trabecular number, *Tb.Sp* Trabecular separation, *ConnD* Connectivity density. Open circles indicate outliers. Horizontal brackets with asterisk (*) indicate significant differences in paired comparisons by using either Tukey test or Dunnett T3 test, as appropriate (*p* ≤ 0.044)

**Fig. 6:**
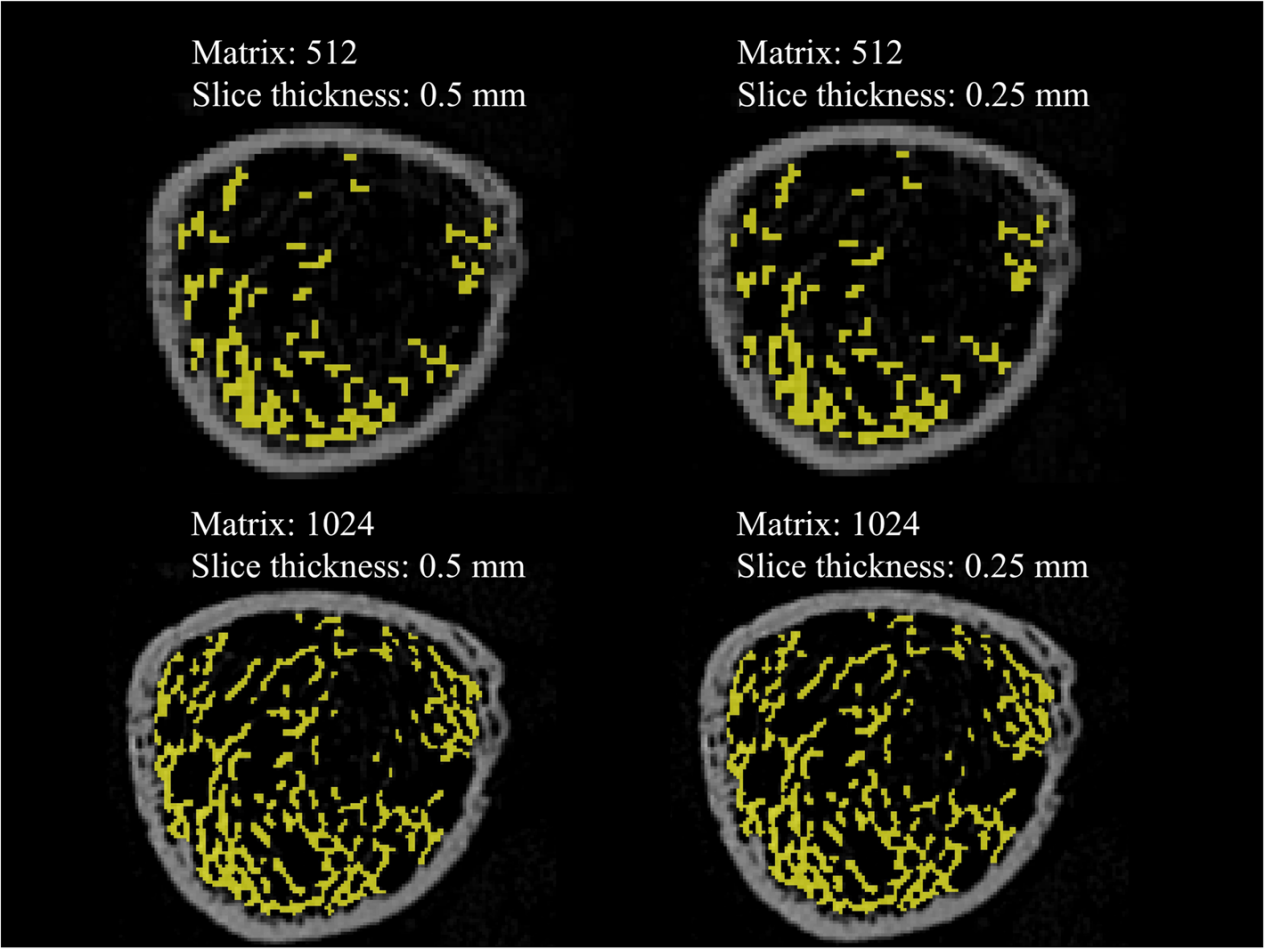
Extracted images representative of the trabecular bone in the human distal tibia on ultra-high-resolution computed tomography (UHRCT). The matrix numbers were 512 × 512 and 1024 × 1024 and the slice thicknesses were 0.5 mm and 0.25 mm

Bone microarchitectural parameters are presented in Table 4 and Fig. 5. vBMD and BV/TV values tended to increase with higher resolutions, especially between different matrix groups; however, these differences were not statistically significant. Tb.Th showed a decreasing trend and statistically significant differences between 512- and 1024-matrix imaging with a slice thickness of 0.5 mm (group H1 *versus* H3, 435.2 μm *versus* 336.6 μm). Tb.N showed an increasing trend and statistically significant differences between imaging procedures with different matrices at both 0.5-mm slice thickness (group H1 *versus* H3, 0.34 l/mm *versus* 0.65 l/mm) and 0.25-mm slice thickness (group H2 *versus* H4, 0.38 l/mm *versus* 0.67 l/mm). Tb.Sp tended to decrease, but the differences were not statistically significant. Like Tb.N, ConnD showed an increasing trend with statistically significant differences between imaging procedures with different matrices at both 0.5-mm slice thickness (group H1 *versus* H3, 0.04 l/mm^3^ *versus* 0.18 l/mm^3^) and 0.25-mm slice thickness (group H2 *versus* H4, 0.06 l/mm^3^ *versus* 0.22 l/mm^3^). Representative extracted images of the trabecular bone are shown in Fig. 6. Changes in matrix numbers yielded more visual differences than changes in slice thickness.

**Table 4:** Comparison of human tibial microarchitectural parameters on UHRCT with different matrix numbers and slice thicknesses *in vivo*

	Group			
	H1	H2	H3	H4
CT scanner	UHRCT	UHRCT	UHRCT	UHRCT
Matrix number	512	512	1024	1024
Slice thickness (mm)	0.5	0.25	0.5	0.25
vBMD (mg/cm^3^)	78.7 ± 47.5	82.6 ± 48.2	104.8 ± 55.9	105.2 ± 56.4
BV/TV (%)	16.9 ± 13.6	17.5 ± 13.6	23.5 ± 14.1	23.8 ± 13.8
Tb.Th (μm)	435.2 ± 114.8	415.2 ± 109.4	336.6 ± 73.4	332.3 ± 72.1
Tb.N (l/mm)	0.34 ± 0.22	0.38 ± 0.24	0.65 ± 0.29	0.67 ± 0.29
Tb.Sp (μm)	5376 ± 6641	4893 ± 6057	1945 ± 2233	1814 ± 2048
ConnD (l/mm^3^)	0.04 ± 0.05	0.06 ± 0.07	0.18 ± 0.11	0.22 ± 0.14

Data are means ± standard deviations

*H1–H4* Human tibia groups 1–4, *UHRCT* Ultra-high-resolution computed tomography, *vBMD* Volumetric bone mineral density, *BV/TV* Trabecular bone volume fraction, *Tb.Th* Trabecular thickness, *Tb.N* Trabecular number, *Tb.Sp* Trabecular separation, *ConnD* Connectivity density

## Discussion

Our study found that microarchitectural parameter trends depended on the *ex vivo* spatial resolution of micro-CT, conventional whole-body CT scanner, and the newly introduced UHRCT, as well as on the slice thickness and matrix numbers for UHRCT. The vBMD, BV/TV, Tb.N, and ConnD parameters tended to increase with increases in resolution both *ex vivo* and *in vivo*. The two highest-resolution UHRCT groups (groups T5 and T6) showed significantly higher values than those obtained in CRCT (group T1) for vBMD, BV/TV, Tb.N, and ConnD. The increasing trend and significant differences between UHRCT and CRCT suggest that the high-resolution feature of UHRCT has better potential for analysing vBMD, BV/TV, Tb.N, and ConnD than does CRCT. Tb.N and ConnD also showed increasing trends, with statistically significant differences between the 512- and 1024-matrix groups *in vivo*. This observation suggests that matrix numbers require more attention than does slice thickness in microarchitectural analysis with UHRCT. In contrast, Tb.Th and Tb.Sp showed a decreasing trend with higher resolutions, except for Tb.Th with micro-CT; however, these differences between UHRCT and CRCT were non-significant.

The microarchitectural parameters showed characteristic trends at various resolutions; vBMD and BV/TV tended to increase with resolution. This trend has been reported previously in a comparison of micro-CT with HR-pQCT [19]. Additionally, our study suggested that this increasing trend was especially prominent in relation to the matrix number; however, these differences were not statistically significant. On the other hand, Tb.N and ConnD showed increasing trends with statistically significant differences between the 512- and 1024-matrix groups *in vivo*.

Previous studies have debated whether higher-resolution imaging increases or decreases the Tb.N value. We found that the mean Tb.N value in human tibiae ranged from 0.34 to 0.67 l/mm. These mean values were lower than those of the tibiae of women with osteoporosis, as reported by a previous study (1.19 l/mm) using HR-pQCT [20]. This further suggests that higher-resolution imaging increases the Tb.N value. Although there have been no reports regarding ConnD, the trends in both ConnD and Tb.N are expected to be similar because Tb.N is estimated as a trabecular bone number and ConnD indicates the number of redundant connections between trabecular structures per unit volume.

Tb.Th and Tb.Sp showed a decreasing trend with higher resolutions, except for Tb.Th on micro-CT. A previous study also reported a decreasing trend in Tb.Th and Tb.Sp on HR-pQCT in comparison with micro-CT [19]. Moreover, the mean values of Tb.Th and Tb.Sp in the tibia of healthy men on HR-pQCT were reported to be 85 μm and 465 μm, respectively [20]; these were lower than the mean values in our study. Tb.Th and Tb.Sp may be reduced by improving the resolution on UHRCT. We speculated that the exception of high Tb.Th values on micro-CT was caused by the low threshold, which was adjusted to detect the trabecular bone on CRCT, since the trabecular bone in the endosteal area on micro-CT could not be distinguished individually (Fig. 3).

We also found that the matrix number affected microarchitectural parameters more strongly than slice thickness. In our *ex vivo* study, 1024- and 2048-matrix imaging with 0.25-mm thickness showed significantly different findings from CRCT with respect to vBMD, BV/TV, and ConnD, but these significant differences did not appear in 512-matrix imaging with 0.5-mm thickness. In particular, Tb.N in 2048-matrix imaging was not different from that of micro-CT. *In vivo*, statistical differences were noted in Tb.Th, Tb.N, and ConnD in relation to matrix number, but none of the parameters showed differences related to slice thickness.

Our study had two main limitations. First, our sample sizes of tiger vertebra and human tibia were small. Ideally, the correlations of microarchitectural parameters in micro-CT and UHRCT should be determined with a larger sample size. However, our preliminary investigations revealed specific trends in microarchitectural parameters at different resolutions. These trends can be used as precise indicators for the depiction of performance according to technical advances such as new iterative reconstruction and deep learning reconstruction techniques. Second, this study also revealed significant differences between UHRCT and micro-CT regarding the vBMD, BV/TV, Tb.Th, and Tb.Sp parameters. Hence, further studies are warranted to determine whether early osteoporosis can be detected with UHRCT. Similarly, the significant differences between whole-body CT, CRCT, and UHRCT have important implications because the whole-body CT enables microarchitectural analysis with imaging ranges as small as 10 mm at any bone site, thus facilitating the precise analysis of microarchitecture.

In conclusion, we observed the characteristic trends of bone microarchitectural parameters at different spatial resolutions. High-resolution imaging in UHRCT demonstrated a better potential for analysing vBMD, BV/TV, Tb.N, and ConnD than did CRCT. Moreover, our study indicated that matrix numbers require more attention than does slice thickness in microarchitectural analysis with UHRCT.

## Data Availability

Not applicable.

## Abbreviations

BMD: Bone mineral density
BV/TV: Trabecular bone volume fraction (bone volume/total volume)
ConnD: Connectivity density
CRCT: Conventional-resolution computed tomography
CT: Computed tomography
DXA: Dual-energy x-ray absorptiometry
HR-pQCT: High-resolution peripheral quantitative CT
Tb.N: Trabecular number
Tb.Sp: Trabecular separation
Tb.Th: Trabecular thickness
UHRCT: Ultra-high-resolution CT
vBMD: Volumetric bone mineral density
